# Profiling of Diagnostic Information of and Latent Susceptibility to Bacterial Keratitis From the Perspective of Ocular Bacterial Microbiota

**DOI:** 10.3389/fcimb.2021.645907

**Published:** 2021-05-13

**Authors:** Zhichao Ren, Qing Liu, Wenfeng Li, Xian Wu, Yanling Dong, Yusen Huang

**Affiliations:** ^1^ Qingdao Eye Hospital of Shandong First Medical University, Qingdao, China; ^2^ State Key Laboratory Cultivation Base, Shandong Provincial Key Laboratory of Ophthalmology, Shandong Eye Institute, Shandong First Medical University & Shandong Academy of Medical Sciences, Qingdao, China; ^3^ Qingdao University Medical College, Qingdao, China; ^4^ Department of Medical Oncology, The Affiliated Hospital of Qingdao University, Qingdao, China

**Keywords:** ocular microbiome, bacterial keratitis, causative bacteria, latent susceptibility, dysbiosis, next-generation sequencing

## Abstract

The ocular surface possesses its own bacterial microbiota. Once given a chance, opportunistic pathogens within ocular microbiota may lead to corneal infection like bacterial keratitis (BK). To reveal the possible factor that makes people vulnerable to BK from the perspective of ocular bacterial microbiota, as well as to compare diagnostic information provided by high-throughput 16S rDNA sequencing and bacterial culture, 20 patients with BK and 42 healthy volunteers were included. Conjunctival swabs and corneal scrapings collected from the diseased eyes of BK patients were subjected for both high-throughput 16S rDNA sequencing and bacterial culture. Conjunctival swabs collected from the normal eyes of BK patients and healthy volunteers were sent only for sequencing. For identifying the pathogens causing BK, high-throughput 16S rDNA sequencing presented a higher positive rate than bacterial culture (98.04% vs. 17.50%), with 92.11% reaching the genus level (including 10.53% down to the species level). However, none of the sequencing results was consistent with the cultural results. The sequencing technique appears to challenge culture, and could be a complement for pathogen identification. Moreover, compared to the eyes of healthy subjects, the ocular microbiota of three sample groups from BK patients contained significantly less *Actinobacteria* and *Corynebacteria* (determinate beneficial symbiotic bacteria), but significantly more *Gammaproteobacteria*, *Pseudomonas*, *Bacteroides*, and *Escherichia-Shigella* (common ocular pathogenic bacteria). Therefore, it is speculated that the imbalance of protective and aggressive bacteria in the ocular microbiota of healthy people may trigger susceptibility to BK. Based on this speculation, it seems promising to prevent and treat infectious oculopathy through regulating ocular microbiota.

## Introduction

The ocular surface possesses its own bacterial microbiota, which contains both probiotics and opportunistic organisms (Huang et al., 2016; St Leger et al., 2017). Once given a chance, such as ocular trauma, opportunistic pathogens within ocular microbiota may lead to devastating corneal infection like bacterial keratitis (BK) (Chidambaram et al., 2018). BK accounts for over 90% of microbial keratitis in temperate regions (Ong and Corbett, 2015). Under a conservative estimate, there are 1.5–2.0 million cases of monocular blindness annually associated with this disease (Shah et al., 2011; Ong and Corbett, 2015; Bograd et al., 2019)

Bacterial culture is considered as the gold standard for etiological diagnosis of BK (Sharma, 2012; Ren et al., 2020). However, this technique is becoming limited in the current diagnostic application for its frequent unsatisfying positive rates (Ren et al., 2020). Culture lacks specificity with its positivity for the healthy ocular surface being as high as 42% to 80% (Willcox, 2013; Huang et al., 2016; Bograd et al., 2019). Sometimes the culture results of specimens from BK patients do not concur with the microscopic examination results of stained smears (Eguchi et al., 2017). Thus, the positive results from bacterial culture may be attributed to certain symbiotic bacteria on the ocular surface that are easy to grow in the culture medium, rather than the exactly real pathogens. A therapeutic schedule of BK usually depends on the antibiotic susceptibility and pathogenicity of pathogenic bacteria, which requires accurate identification of specific pathogens (Bourcier et al., 2003).

The Human Microbiome Project was initiated by the National Institutes of Health in 2007 to identify the microorganisms that reside normally on the healthy human body and to ultimately characterize changes associated with disease states (Integrative HMPRNC, 2019). The investigate interactions between the gut microbiota and ocular pathology and their implications for progression of disease, namely “gut-eye” axis, has been focusing on for a long time (Kugadas et al., 2017; Floyd and Grant, 2020). However, the breakthrough related to the reciprocity between ocular microbiota and eye were not available until St. Leger et al. isolated *Corynebacterium mastitidis*, a type of ocular probiotics, on bacterial culture for the first time in 2017 (St Leger et al., 2017). Thereupon, for healthy people, whether ocular microbiota generally maintains a dynamic equilibrium status and whether dysbiosis of ocular microbiota can facilitate oculopathy become necessary to be explored, especially through new technologies and approaches.

Since the 16S rDNA gene not only has highly conserved structure and function but also can reflect the differences among different bacterial genera, it is generally used as a unique barcode for bacterial identification (Wang et al., 2007). High-throughput 16S rDNA sequencing has opened a new avenue for thorough comprehension of the human bacterial microbiome, for it can exhibit the relative content of almost all bacterial genera in a sample. In this study, from the perspective of ocular bacterial microbiota, the diagnostic information within the ocular bacterial microbiome for identifying causative bacteria of BK was disclosed using this sequencing technique, and the susceptibility factor of BK was revealed.

## Materials and Methods

### Ethics Approval

This research was approved by the Ethics Committee of Qingdao Eye Hospital and registered on Chinese Clinical Trial Registry (ChiCTR 1900023651). All procedures complied with the tenets of the Declaration of Helsinki. Informed consent was obtained from all participants.

### Inclusion Criteria

Subject recruitment lasted for one whole year to eliminate seasonal effects. Twenty patients, 10 males and 10 females, who were diagnosed as BK following the Bacterial Keratitis Preferred Practice Pattern from the American Academy of Ophthalmology (Lin et al., 2019) were included, with an average age of 50 years (range, 24 to 80 years). Patients with any recent diagnosis and treatment in other eye care institutions, recent usage of eyedrops by themselves, concurrent eye diseases, and any previous oculopathy or ophthalmic/corneal refractive surgery were excluded. Based on the stratified sampling, 42 healthy volunteers with a similar gender and age composition were enrolled as controls.

### Sample Collection

In a disinfected operating room utilizing a laminar flow system (conforming to GB50333-2013-I standard of China), all participants lay supine for collecting corneal scrapings and/or conjunctival samples. Eye lids were wiped using Iodophor, and the remaining facial area was covered with a sterile surgical drape. The palpebral, bulbar, and fornical conjunctiva of the affected and normal eyes of each patient as well as the randomly chosen eye of each healthy control was rubbed lightly using a sterile swab soaked with oxybuprocaine hydrochloride eye drops for preparation of conjunctival samples. Then, after a drop of oxybuprocaine hydrochloride eye drops was dripped to the diseased eyes, the corneal lesions were scraped using an ophthalmic microsurgical knife (Cat. No. MR-G137A, Suzhou Mingren Medical Equipment Co., Suzhou, China) under a microscope.

To avoid potential contamination, after intraday sampling, three sterile swabs exposed to laminar flow for 3 minutes and all remaining oxybuprocaine hydrochloride eye drops were sent for DNA extraction and high-throughput 16S rDNA sequencing. Intraday samples from subjects were kept in reserve so long as aforementioned environmental samples generated negative results.

### Bacterial Culture

Samples were streaked on blood agar medium (Cat. No. 16, Autobio, Zhengzhou, China) at 37°C using the quad plate streaking method, with daily observation. Once macroscopic colonies appeared in the medium, they were stained with Gram staining and verified according to morphology under a microscope by two experienced technicians. Then the colonies were sent into MicroScan WalkAway 96Plus (Siemens, Germany) to identify the genus or species. If no colony formed within 14 days, the culture was reported to be negative.

### High-Throughput 16S rDNA Sequencing

#### DNA Extraction

Total genomic DNA was extracted from samples using the DNA Extraction Kit (Cat. No. D3096-100T, Omega Bio-tek, Norcross, GA, USA), after which all DNA samples were delivered to OE Biotech (Qingdao, China) for high-throughput 16S rDNA sequencing. All the sequencing and analytic process was subjected to the standard operating procedure of OE Biotech. More experimental details about sequencing are available at https://www.qdoebiotech.com.

#### DNA Amplification

After the quantity and quality of DNA were assessed by NanoDrop and agarose gel, samples were loaded into 0.6% agarose gel and subjected to 120V constant voltage electrophoresis for 15 minutes. A total of 50 ng DNA without degradation or with slight degradation were used for PCR amplification (Cat. No. 580BR10905, Bio-rad, Hercules, CA, USA) after being diluted to 1 ng/μl as template, with primers and Takara Ex Taq (Cat. No. RR001Q, Takara, Shiga, Japan), following instructions. V3-V4 variable regions of 16S rRNA were amplified with universal primers 343F (5’- TACGGRAGGCAGCAG -3’) and 798R (5’- AGGGTATCTAATCCT-3’).

#### Library Construction

Amplicon quality was evaluated using gel electrophoresis, refined with AMPure XP beads (Agencourt), and enlarged for another round of PCR using primers 343F and 798R. After purification with AMPure XP beads again, the amplicon was quantified using Qubit dsDNA assay kit (Cat. No. Q32852, Life Technologies, Carlsbad, CA, USA) to become the final amplicon. Equal amounts of refined amplicon were pooled for subsequent high-throughput sequencing (Illumina miseq pe300).

#### Bioinformatic Analysis

Raw sequencing data were stored as FASTQ format. Paired-end reads were operated using Trimmomatic software (version 0.53) to discover and cut out ambiguous bases (N). Trimmomatic software also cut off inferior quality sequences with an average quality score below 20 through the sliding window trimming approach. Paired-end reads were assembled using FLASH software (version 1.2.11) after trimming. Parameters of the assembly were set as 10 bp of minimal overlapping, 20% of maximum mismatch rate, and 200 bp of maximum overlapping. Moreover, sequences were further denoised by quitting reads with ambiguous, homologous sequences or those below 200 bp. Reads with 75% of bases beyond Q20 were reserved, while those having chimera were removed. The two processes were performed using QIIME software (version 1.8.0). Clean reads were further subjected to primer sequences removal and clustering to form operational taxonomic units (OTUs) with 97% similarity cut out using Vsearch software (version 2.4.2). All representative reads of every OTU were chosen using QIIME package. All representative reads were annotated and blasted against Silva database (version 123) and Greengens database using RDP classifier (confidence threshold was 70%).

Using the statistical software R (corrplot package), a predicted interaction network of top 30 bacterial genera was established based on Spearman correlation coefficient. The bacterial genera with |SpearmanCoef| > 0.8 and p < 0.01 were highlighted.

### Parameters of Microbial Community

Alpha diversities were used to delineate within-community characteristics. Good’s coverage index reflected the depth of sequencing. The closer the index was to 1, it meant that the depth of sequencing had basically covered all species in the sample. Observed species parametrized the actual number of observed OTUs. Chao1 index estimated how many kinds of OTUs actually presented in the community (Hill et al., 2003). Both Shannon Wiener index and Simpson’s index of diversity (1-D) are estimators of species richness and evenness, but the former was more sensitive to species richness, whereas the latter was more sensitive to evenness (Hill et al., 2003; Kim et al., 2017). The larger the value of phylogenetic diversity index represented the microbial community was constituted by species that had farther relationship of evolution between each other (Faith and Baker, 2007). Moreover, beta diversities were used to reveal between-community characteristics in order to compare the differences among the diseased eyes and normal eyes of BK patients and the eyes of healthy volunteers.

## Results

### Overview of the Study

**Figure 1:**
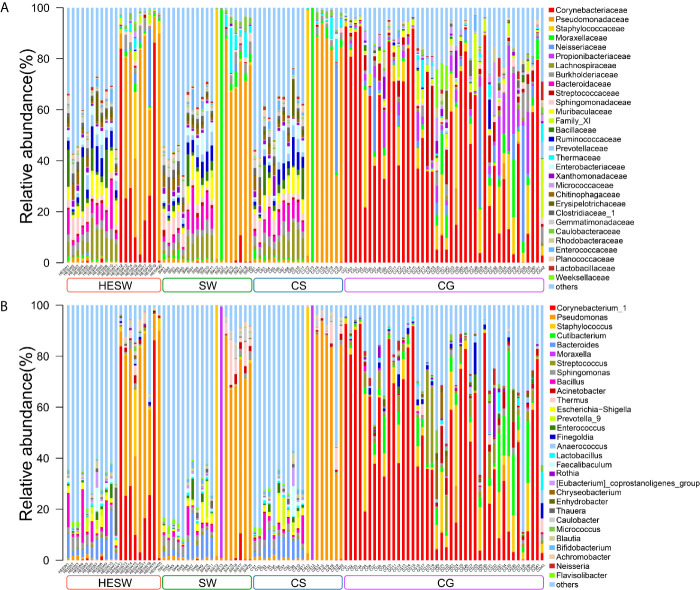
The relative composition of ocular microbiota. Each bar represents a sample of conjunctival microbiota, various color portions represent different genera, and the length of a colored portion represents the relative content of a family **(A)** or genus **(B)**. Among the 102 samples, 100 positive samples were obtained, including 20 conjunctival swabs from the healthy eyes of patients with bacterial keratitis (BK) (group HESW), 19 conjunctival swabs from the diseased eyes of BK patients (group SW), 19 corneal scrapings from the diseased eyes of BK patients (group CS), and 42 conjunctival swabs from the healthy volunteers (group CG). Different samples possess diverse ocular surface microbiotas.

With high-throughput 16S rDNA sequencing, 98.04% of specimens (100/102) presented positive results, including 19 conjunctival swabs (95%, 19/20) and 19 corneal scrapings (95%, 19/20) from the diseased eyes, 20 conjunctival swabs (100%, 20/20) from the healthy eyes of BK patients, and 42 conjunctival swabs (100%, 42/42) from the eyes of healthy volunteers. The amount of OTUs in each sample ranged from 10 to 3666. The content and the representative sequence of each OTU in each sample are shown in **Supplementary Data 1, 2**. The relative bacterial compositions of top 30 families and genera for each sample are presented in **Figure 1**.

On the other hand, the results of bacterial culture were positive in 17.5% of samples (7/40) from the diseased eyes, including 2 conjunctival swabs and 5 corneal scrapings. In total, 4 bacterial genera were found (**Table 1**).

**Table 1:** The diagnostic information of high-throughput 16S rDNA sequencing and bacterial culture.

No.	Gender	Age	Conjunctival swabs		Corneal scrapings		Whether two sequencing results is same	Whether sequencing results same to culture
			16S rDNA sequencing	Culture	16S rDNA sequencing	Culture		
1	Female	61	f_Enterobacteriaceae g_Escherichia-Shigella (2.5%)	–	f_Moraxellaceae (2.3%)	–	No	No
2	Female	69	g_Sphingomonas (1.5%)	–	g_Sphingomonas (1.6%)	g_Micrococcus	Yes	No
3	Male	80	g_Solanum torvum s_Solanum torvum (3.3%)	–	g_Bacillus (6.7%)	–	No	No
4	Male	52	f_Moraxellaceae (2.7%)	f_Enterobacteriaceae g_Proteus s_Proteus mirabilis	f_Bacteroidaceae g_Bacteroides s_Bacteroides vulgatus (5.2%)	f_Enterobacteriaceae g_Proteus s_Proteus mirabilis	No	No
5	Female	65	g_Alloprevotella (1.2%)	–	g_Pseudoxanthomonas (5.9%)	–	No	No
6	Female	38	g_Bacillus (7.2%)	–	g_Escherichia-Shigella (10.7%)	–	No	No
7	Male	35	g_Enterococcus (8.1%)	–	g_Prevotella (11.0%)	–	No	No
8	Male	66	g_Escherichia-Shigella (2.2%)	–	g_Bacteroides (3.5%)	g_Streptococcus s_Streptococcus pneumoniae	No	No
9	Male	67	g_Escherichia-Shigella (7.3%)	–	g_Faecalibaculum (11.4%)	–	No	No
10	Male	67	g_Prevotella (13.2%)	–	g_Escherichia-Shigella (4.1%)	–	No	No
11	Female	67	g_Escherichia-Shigella (3.2%)	–	g_Bacillus (8.2%)	–	No	No
12	Female	51	g_Staphylococcus s_Staphylococcus warneri (97.9%)	–	g_Staphylococcus s_Staphylococcus warneri (96.3%)	–	Yes	No
13	Female	73	g_Moraxella (98.5%)	g_Staphylococcus s_Staphylococcus epidermidis	g_Moraxella (99.6%)	g_Staphylococcus s_Staphylococcus epidermidis	Yes	No
14	Male	57	g_Pseudomonas (86.8%)	–	g_Pseudomonas (88.3%)	–	Yes	No
15	Male	24	g_Pseudomonas (64.4%)	–	g_Pseudomonas (92.2%)	g_Staphylococcus s_Staphylococcus epidermidis	Yes	No
16	Male	43	g_Pseudomonas (65.5%)	–	g_Pseudomonas (87.6%)	–	Yes	No
17	Female	67	g_Pseudomonas (67.5%)	–	–	–	No	No
18	Female	80	g_Pseudomonas (69.4%)	–	g_Pseudomonas (82.1%)	–	Yes	No
19	Male	63	–	–	f_Neisseriaceae (42.3%)	–	No	No
20	Female	45	g_Pseudomonas (75.1%)	–	g_Pseudomonas (82.0%)	–	Yes	No
Positive rate (coincidence rate)			95%	10%	95%	25%	40%	0%

f, at the family level; g, at the genus level; s, at the species level; the percentage in brackets represents the relative content of the dominant species.

### Identification of the Pathogenic Bacteria

All high-throughput 16S rDNA sequencing reports disclosed 3 results at the family level, 31 results at the genus level, and 4 results at the species level. Meanwhile, bacterial culture exposed 1 result at the genus level and 6 results at the species level. However, none of the sequencing results was consistent with the culture results (**Table 1**). The positive rate of high-throughput 16S rDNA sequencing was higher than bacterial culture (98.04% vs. 17.5%), but the sequencing presented a lower percentage of results at the genus level (92.11% vs. 100%).

Using high-throughput 16S rDNA sequencing, as shown in **Table 1**, 18 patients obtained positive results from both conjunctival swabs and corneal scrapings of the diseased eyes. The 18 pairs of results can be analyzed according to two hypotheses. If the genus with the highest relative content was hypothesized as the result for identifying pathogens, the sequencing results from conjunctival swabs and corneal scrapings were consistent in eight of 18 patients (44.44%). If the genus with the highest relative content was deemed the causative bacterium as long as it occupied more than 50% of the content and other conditions were deemed multiple bacterial co-infections, all results (100%, 18/18) from conjunctival swabs and corneal scrapings were consistent. Thus, in the latter circumstance, conjunctival swabs seemed to be an alternative noninvasive approach to etiological examination of BK.

### Community Structural Alterations

Due to the similar environmental stress, the right and left eyes have no difference in the structure of microbiota (Cavuoto et al., 2018). If an infection in one eye would not lead to an alteration of the bacterial community of the other eye, the microbiota of the healthy eyes of BK patients exactly reflected the status before BK occurred. Based on this hypothesis, the comparison between the conjunctival swabs from the healthy eyes of BK patients and the normal volunteers could reveal the susceptibility factor of BK from the perspective of ocular microbiota.

**Figure 2:**
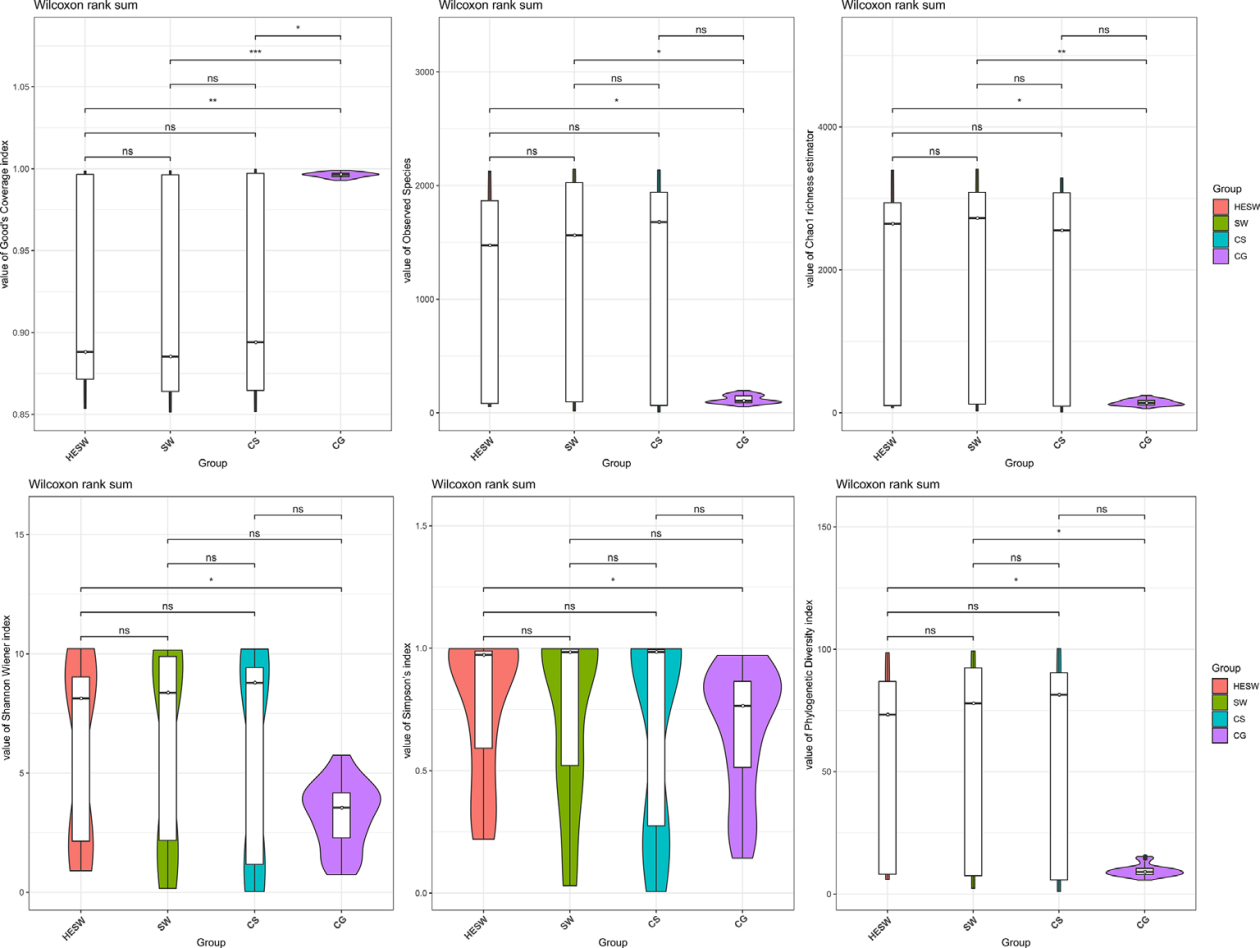
On the dimensionality of alpha diversities, six indices all suggest the sort and richness of ocular bacterial microbiota from the healthy eyes of BK patients had significant difference from the eyes of healthy people. Alpha diversity indices in this study includes Good’s coverage index, Observed species, Chao1 index, Shannon Wiener index, Simpson’s index, and phylogenetic diversity index. (HESW, conjunctival swabs from the healthy eyes of BK patients; SW, conjunctival swabs from the diseased eyes of BK patients; CS, corneal scrapings from the diseased eyes of BK patients; CG, conjunctival swabs from healthy volunteers; ns, no significant difference; *P < 0.05; **P < 0.01; ***P < 0.001).

**Figure 3:**
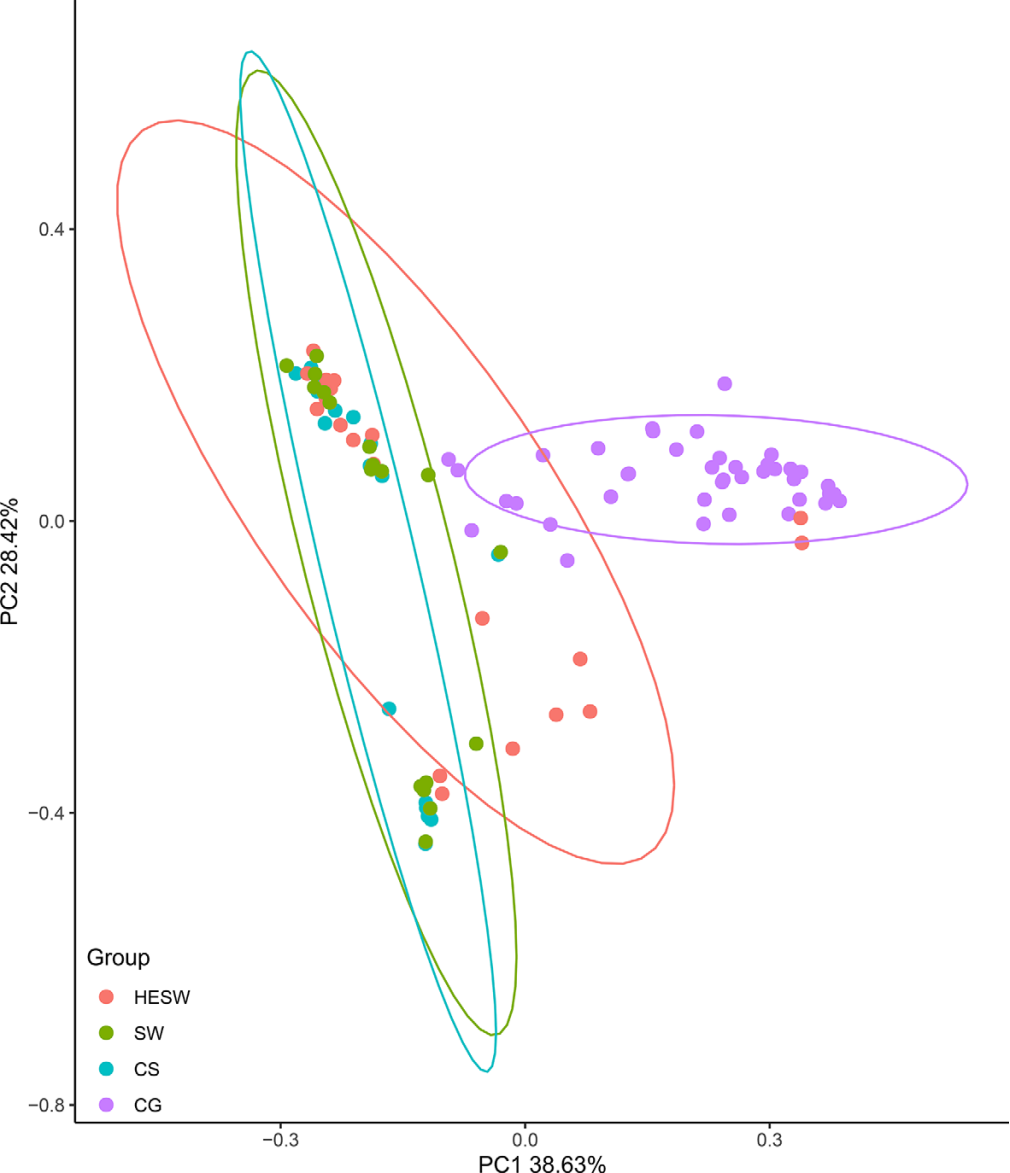
On the dimensionality of beta diversities, PCoA plots suggest the sort and richness of ocular bacterial microbiota from the healthy eyes of BK patients had significant difference from the eyes of healthy people. Two-dimensional PCoA plots with confidence ellipse (Weighted Unifrac algorithm) show the beta diversities of each sample. The abscissa (PC1) and the ordinate (PC2) are the two principal co-ordinates which have the largest interpretation of the difference between samples. The percentage on abscissa (or ordinate) represents the explanatory degree to the difference among samples. The CG group had significant difference from the HESW group (HESW, conjunctival swabs from the healthy eyes of BK patients; SW, conjunctival swabs from the diseased eyes of BK patients; CS, corneal scrapings from the diseased eyes of BK patients; CG, conjunctival swabs from the healthy volunteers; BK, bacterial keratitis).

With respect to alpha diversities (**Figure 2**), the healthy eyes of BK patients were significantly different from the healthy eyes of controls in Good’s coverage index (P=0.00145), observed species (P=0.044), Chao1 index (P=0.0276), Shannon Wiener index (P=0.039), Simpson’s index (P=0.025), and phylogenetic diversity index (P=0.012). All were of pairwise comparisons using Wilcoxon rank sum test. For beta diversity (**Figure 3**), the PCoA plots (Weighted Unifrac algorithm, P=0.001, R2 = 1, F. Model=13.823, total SumsOfSqs=12.1678, and total Df=99) showed significant difference between the healthy eyes from the patients and the controls. On the whole, the sort and richness of ocular bacterial microbiota of the healthy eyes of BK patients had significant difference from the eyes of healthy people.

**Figure 4:**
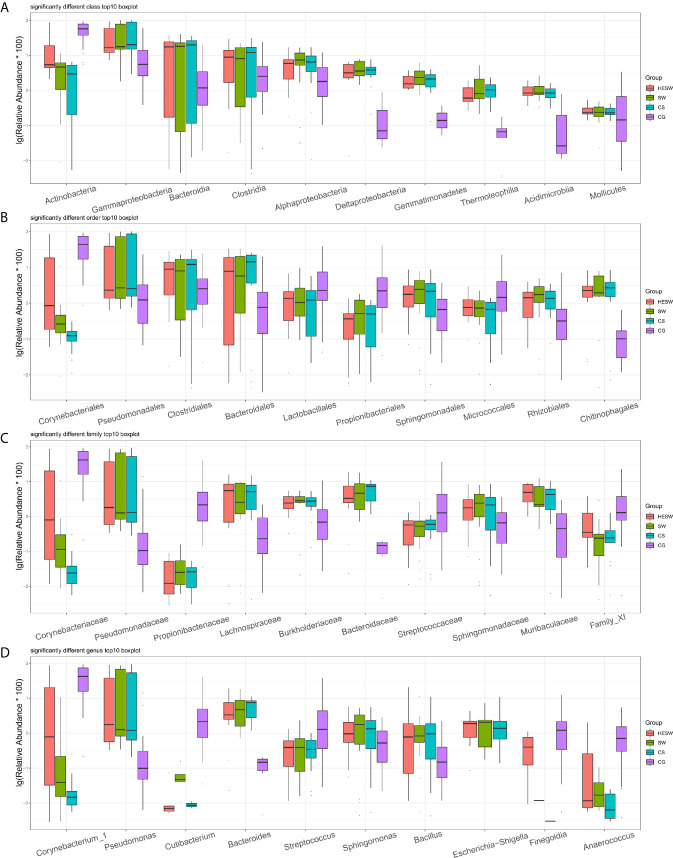
Top 10 taxa with significant difference at the levels of class, order, family, and genus. **(A)** At the class level, *Actinobacteria* significantly decreased and *Gammaproteobacteria* significantly increased in BK patients’ ocular microbiota. **(B–D)** At the levels of order, family, and genus, *Corynebacter* significantly decreased, while *Pseudomonas*, *Bacteroides*, and *Escherichia-Shigella* significantly increased in BK patients’ ocular microbiota (HESW, conjunctival swabs from the healthy eyes of BK patients; SW, conjunctival swabs from the diseased eyes of BK patients; CS, corneal scrapings from the diseased eyes of BK patients).

**Figure 4** shows the top 10 taxa with significant difference (p values were shown in **Table S1**) among four groups at the levels of class, order, family, and genus. Compared to the healthy subjects, the ocular microbiota of the healthy eyes of BK patients possessed less *Actinobacteria*, but more *Gammaproteobacteria*, *Bacteroidia*, *Clostridia*, *Alphaproteobacteria*, *Deltaproteobacteria*, *Gemmatimonadetes*, *Thermoleophilia*, *Acidimicrobiia*, and *Mollicutes* at the class level; less *Corynebacteriales*, *Lactobacillales*, *Propionibacteriales*, and *Micrococcales*, but more *Pseudomonadales*, *Clostridiales*, *Bacteroidales*, *Sphingomonadales*, *Rhizobiales*, and *Chitinophagales* at the order level; less *Corynebacteriaceae*, *Propionibacteriaceae*, *Streptococcaceae*, and *Family XI*, but more *Pseudomonadaceae*, *Lachnospiraceae*, *Burkholderiaceae*, *Bacteroidaceae*, *Sphingomonadaceae*, and *Muribaculaceae* at the family level; less *Corynebacterium*, *Cutibacterium*, *Streptococcus*, *Finegoldia*, and *Anaerococcus*, but more *Pseudomonas*, *Bacteroides*, *Sphingomonas*, *Bacillus*, and *Escherichia-Shigella* at the genus level.

**Figure 5:**
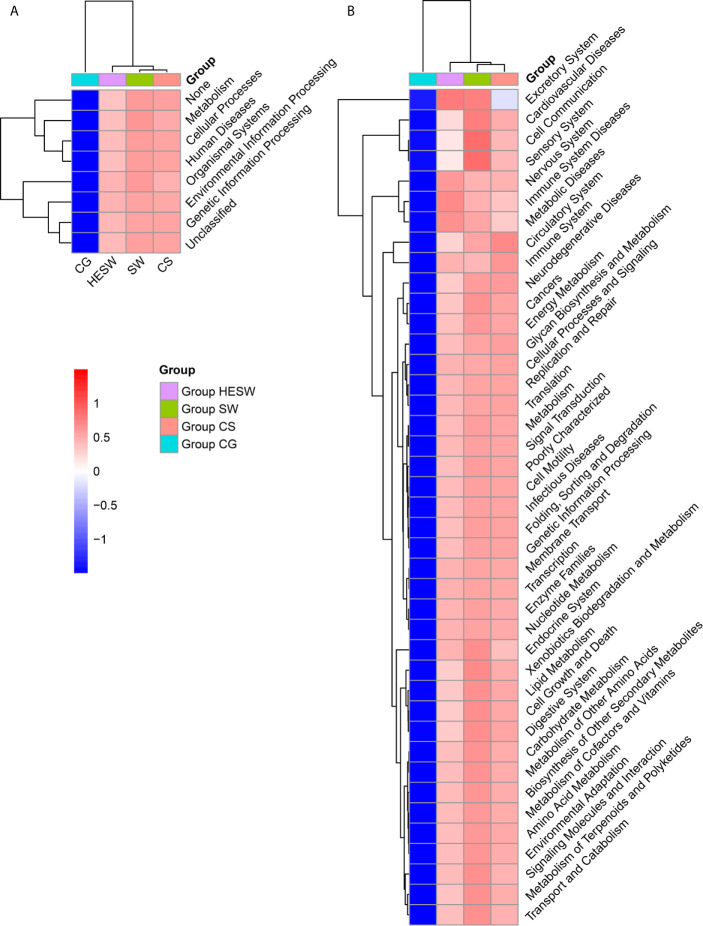
The ocular microbiota of BK patients appeared more active and aggressive. At L1 **(A)** and L2 **(B)** level, two-dimensional heat map showing rank normalized abundances (scaled between 0 and 1) of differentially abundant bacterial gene functions determined by Kruskal-Wallis test and KEGG. (HESW, conjunctival swabs from the healthy eyes of BK patients; SW, conjunctival swabs from the diseased eyes of BK patients; CS, corneal scrapings from the diseased eyes of BK patients).

Based on PICRUSt (Langille et al., 2013), the ocular microbiota of three sample groups from BK patients were found to possess significantly different composition of bacterial gene functions, compared to the eyes of healthy subjects. Compared to the healthy subjects, the ocular microbiota of BK patients presented significantly more gene related to metabolism, cellular processes, human diseases, organismal systems, environmental information processing, and genetic information processing (**Figure 5**). Namely, the ocular microbiota of BK patients appeared more active and aggressive.

### A Predicted Interaction Network of Ocular Bacterial Microbiota at the Genus Level

Based on Spearman correlation coefficient (|SpearmanCoef|> 0.8 and P <0.01) of the top 50 bacterial genera, **Figure S1** reveals a predicted higher-order interaction network at the genus level for ocular bacterial microbiota. This network was not verified *in vitro*, but it still may help to enlighten further investigations.

## Discussion

### High-Throughput 16S rDNA Sequencing Challenges Bacterial Culture in Identifying the Pathogenic Bacteria of BK

Despite the worldwide recognition of culture in identifying microorganisms responsible for corneal infection (Austin et al., 2017; Eguchi et al., 2017), it has been facing an increasing challenge. According to our previous studies, microbiota abounding on the ocular surface of both healthy people and patients with non-infective eye disease undermines the reliability of microbial culture (Huang et al., 2016; Dong et al., 2019; Ge et al., 2019; Wang et al., 2019; Ren et al., 2020). High-throughput internal transcribed spacer (the unique barcode for fungal identification) sequencing also presents advantages over fungal culture for fungal identification in fungal keratitis (Ren et al., 2020). The inconformity of results between culture and high-throughput 16S rDNA sequencing seems to be within expect, but it does not mean high-throughput sequencing technology could supersede microbial culture, for sequencing has deficiencies like PCR bias, insufficient coverage for microorganisms of public sequence databases, and incapability to distinguish resident and transient microorganisms, living microorganisms and simply DNA signatures (Nilsson et al., 2008; Shivaji et al., 2019). Furthermore, high-throughput 16S rDNA sequencing technology cannot test antibiotic susceptibility, which is important for treating BK. Methodologically, high-throughput 16S rDNA sequencing requires amplification step, however, culture have no need for amplification. Therefore, the amplification step also may account for the discrepancy in the numbers/types of revealed organisms. After weighing the pros and cons in this study, high-throughput 16S rDNA sequencing was considered to be an optional supplement for identification of bacterial pathogens in the diagnosis of BK.

It is hard to determine which result we should believe when divergent results are generated after sequencing and culture. In the current study, high-throughput 16S rDNA sequencing showed that the causative genus occupied more than 50% of ocular bacterial microbiota only when it was identified as *Staphylococcus*, *Moraxella*, and *Pseudomonas*, while in other situations it usually occupied less than 15% (scilicet multiple bacterial co-infections). This phenomenon was not in accordance with traditional cognition for infectious disease – a certain causative genus wildly grows and occupies absolute quantitative superiority. It seems that the causative genus of BK, along with its antibiotic resistance, should be comprehended anew.

One of the possible new understandings for BK may be similar to the revolution of comprehensions for bacterial vaginosis (BV). BV was first described to be caused by *Haemophilus vaginalis* or *Gardnerella vaginalis* (Ledger, 1993). Then it was considered to be related to a number of potential microbial pathogens, singly and in combinations (Onderdonk et al., 2016). Finally, BV was attributed to the reduction of *Lactobacillus* and the overgrowth of anaerobic bacteria (Redelinghuys et al., 2020). This eventuality reminds us of the probability of regulating one certain species or a category of species, instead of concentrating on the causative species, in the treatment of BK.

### The Imbalance of Protective and Aggressive Bacteria in the Ocular Microbiota for Healthy People Triggers Susceptibility to BK

The ocular microbiota, which comprised both probiotic and pathogenic bacteria (**Figure 1**), and various host cells were disclosed to process a complicated interaction network in our study (**Figure S1**). The ocular surface microenvironment might be explained by the Yin-Yang balance, which is a basic theory in the traditional Chinese medicine, as well as a philosophical term. Therein Yin is on behalf of the inhibitory and repressive factors, whereas Yang represents the aggressive and active factors (Yan et al., 2020). Normally, Yin and Yang maintain a dynamic equilibrium status. However, Yin-Yang imbalances enable people to develop certain disease. Accordingly, in this study, Yin stood for probiotics such as *Corynebacterium* and *Actinobacteria*, for *Corynebacterium mastiditis* (St Leger et al., 2017) has been previously demonstrated to elicit IL-17 response from γδ T cells in the ocular mucosa to resist bacterial and fungal infections on the murine ocular surface, and *Actinobacteria* (Valliappan et al., 2014; Dalitz et al., 2017) has proved to synthesize a series of secondary metabolites, which have antimicrobial, anti-viral, antiparasitic, antioxidant, anticancer, and neurological activities. Yin also represented a bunch of nonpathogenic bacteria with undiscovered benefits. On the other hand, Yang stood for a series of pathogenic bacteria, such as *Pseudomonas*, *Bacteroides*, *Streptococcus*, and *Escherichia-Shigella*. The ocular microbiota of BK patients was found to be composed of fewer protective bacteria but more aggressive bacteria than that of healthy people. Namely, the Yin-Yang imbalance made the cornea vulnerable.

It is worth noting that Yang does not mean relevant elements are always detrimental in Chinese philosophy. It is also crucial for the complete wholeness of the dynamic equilibrium status. In addition, a Yang element in one certain field can be the Yin in other fields. For example, *Staphylococcus epidermidis* is not only an opportunistic pathogen on the skin but also can produce staphylococcal lipoteichoic acid through Toll-like receptor 3 to impact keratinocytes, eventually inhibiting inflammation after skin injury (Lai et al., 2009). *Corynebacterium mastiditis* is an important pathogen of mastitis, but it can protect the cornea against infection (St Leger et al., 2017)

Moreover, the same inducement can cause diverse types of infection. For instance, corneal trauma may lead to BK, fungal keratitis or *Acanthamoeba* keratitis (Mascarenhas et al., 2014). In addition, lack of the same beneficial bacterial genus can make people vulnerable to diverse diseases. For example, the murine corneas lacking *Corynebacterium mastiditis* were observed to be liable to *Candida albicans* and *Pseudomonas aeruginosa* infections (St Leger et al., 2017). Similarly, our previous study on the human ocular microbiota showed a lower relative abundance of *Corynebacterium* in eyes with fungal keratitis when compared with the normal eyes (Ge et al., 2019). That is, the susceptibility factors of BK may not be on account of the fluctuation of content for simply a certain bacterial genus, but the holistic Yin-Yang balance.

High-throughput sequencing technology provides a holistic horizon for the ocular microbiota. However, the network within the microbiota community and between the microbiota and host cells needs to be eventually verified based on culture technology. Microbiota information combined with other omics technologies, such as metagenomics, metatranscriptomics, metaproteomics and metabolomics, can predict suitable conditions for culture of hard-to-culture microorganisms (Vilanova and Porcar, 2016). Therefore, the combination of cultivation-dependent and cultivation-independent approaches could be helpful. Ziesack et al. (2019) have illuminated the interaction in a conglomerate composed of four bacterial species and have artificially regulated the microbiota structure based on bacterial metabolites. When the network relationship is thoroughly deciphered, the prevention and treatment of oculopathy through regulation of ocular microbiota becomes promising.

Shivaji et al. (2021) recently published similar study. Some of their patients had received recently antibiotic treatment, and a few healthy controls had undergone photorefractive keratectomy. Despite the difference in the inclusion criteria, our study both reported that *Actinobacteria* was significantly reduced in the ocular microbiota of diseased eyes of BK patients when compared with healthy subjects on examinations of conjunctival swabs and corneal scrapings. Compared with their study, this presented study involved additional conjunctival swabs from the normal eyes of BK patients. This may bridge the ocular microbiota before the occurrence and during the course of BK for BK patients. As a consequence, we focused more on the normal eyes of patients and healthy volunteers for exploration of ocular microbiota. Based on the deductive alteration of ocular microbiota before and after the development of BK, we deduced for the first time that the imbalance of protective and aggressive bacteria in the ocular microbiota may make eyes vulnerable to BK.

This study has limitations. Although volunteers had been recruited for a long period of time, the amount of participants was still limited. The abundances of bacteria cannot be quantitative analyzed because the process of high-throughput sequencing involved amplification steps. Furthermore, the reason for the imbalance of ocular microbiota was not ascertained. Since similar microbiotas can generate different interrelations and functions (Heintz-Buschart et al., 2016), further investigations on the regulation of the structure of ocular microbiota, metabolism, and antibiotic resistance would be made based on multi-omics technology. Besides, aberrations in the gut microbiota have been provided to be associated with ocular diseases in both human and animal studies (Floyd and Grant, 2020), however, this study did not explore the interaction between gut microbiota and BK.

## Conclusion

High-throughput 16S rDNA sequencing challenged bacterial culture and could be a complement in identifying bacterial pathogens in corneas. Moreover, this study suggests the imbalance of protective and aggressive bacteria of ocular microbiota may trigger susceptibility to BK in humans. Regulation of ocular microbiota seems to be promising for prevention and treatment of ocular disease.

## Data Availability Statement

The datasets presented in this study can be found in online repositories. The names of the repository/repositories and accession number(s) can be found below: NCBI BioProject, accession no: PRJNA692666.

## Ethics Statement

The studies involving human participants were reviewed and approved by the Ethics Committee of Shandong Eye Institute. The patients/participants provided their written informed consent to participate in this study.

## Author Contributions

ZR contributed to literature search, data analysis and drafting of the manuscript. QL contributed to the conjunctival swab collection and patient management. WL contributed to the design of this research and revision of the manuscript. XW contributed to literature search and data analysis. YD contributed to the clinical diagnosis. YH contributed to the design of this research, literature search, data analysis and revision of the manuscript. All authors contributed to the article and approved the submitted version.

## Funding

This research was supported by the National Natural Science Foundation of China (grant number 81970788), the Key Science and Technology Innovation Project of Shandong Province (grant number 2018CXGC1205), and the Taishan Scholar Program (grant number ts20190983).

## Conflict of Interest

All authors declare that the research was conducted in the absence of any commercial or financial relationships that could be construed as a potential conflict of interest.
