# Supplementary material for: Profiling of Diagnostic Information of and Latent Susceptibility to Bacterial Keratitis From the Perspective of Ocular Bacterial Microbiota
Source: Front Cell Infect Microbiol. 2021 May 13;11:645907. doi: 10.3389/fcimb.2021.645907 (PMC8155582; doi:10.3389/fcimb.2021.645907)
Supplement: Supplementary file 1 [file DataSheet_1.docx]

Supplementary Material

# Supplementary Data

Supplementary data 1: The amount of tags of diverse OTUs for various samples.

Supplementary data 2: Representative sequences and classification annotations of various OTUs.

# Supplementary Figures and Tables

## Supplementary Figures


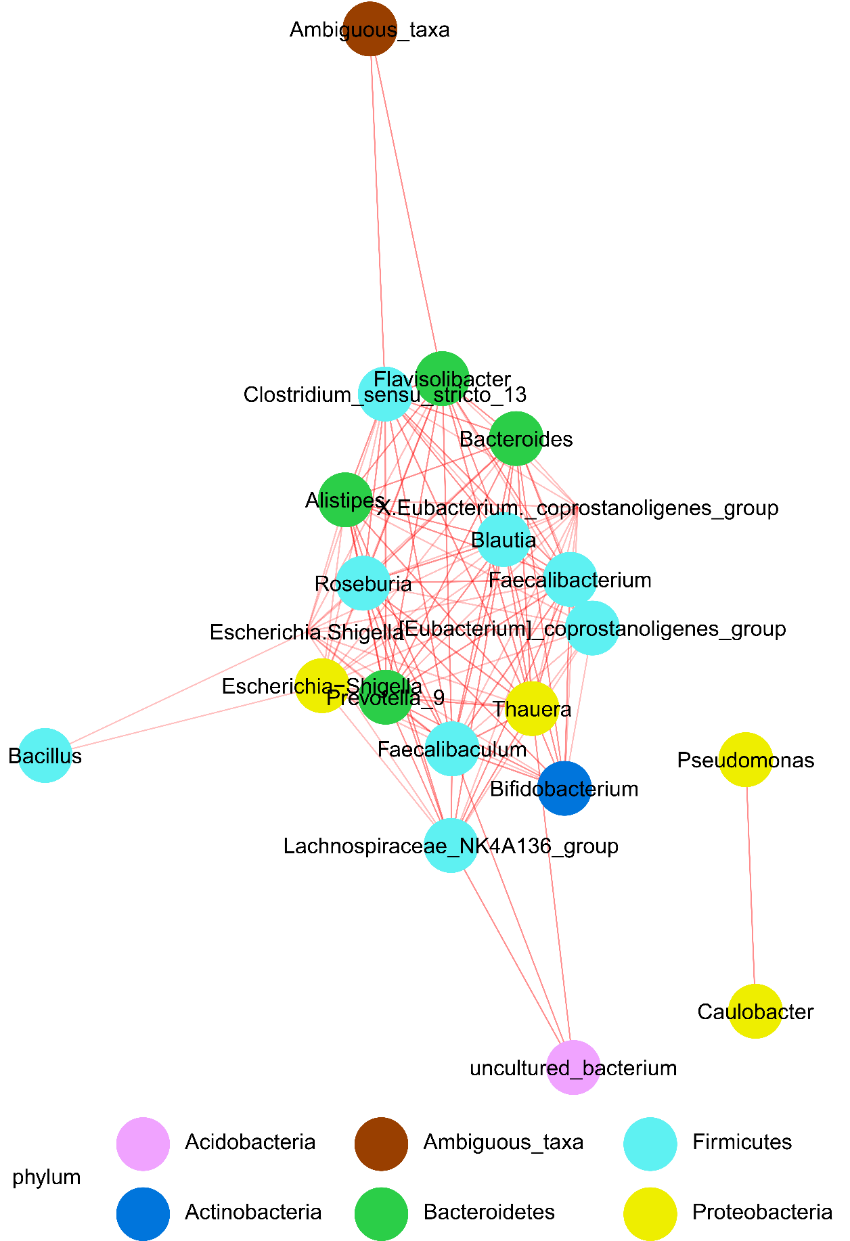


**Supplementary Figure 1.** **A predicted interaction network of ocular bacterial microbiota at the genus level.** Based on Spearman correlation coefficient (|SpearmanCoef|> 0.8 and P <0.01) of top 50 bacterial genera, the interaction network was speculated among various bacterial genera. Red lines represent positive correlations.

## Supplementary Tables

**Supplementary Table 1. P values of top 10 taxa with significant difference at the levels of class, order, family, and genus.**

| **Class** | **P value** | **Order** | **P value** | **Family** | **P value** | **Genus** | **P value** |
| --- | --- | --- | --- | --- | --- | --- | --- |
| Actinobacteria | 1.11E-16 | Corynebacteriales | 5.74E-13 | Corynebacteriaceae | 8.73E-13 | Corynebacterium_1 | 1.53E-12 |
| Gammaproteobacteria | 3.21E-05 | Pseudomonadales | 4.16E-04 | Pseudomonadaceae | 6.32E-04 | Pseudomonas | 6.41E-04 |
| Bacteroidia | 2.69E-05 | Clostridiales | 2.61E-02 | Propionibacteriaceae | 1.33E-04 | Cutibacterium | 1.42E-04 |
| Clostridia | 2.47E-02 | Bacteroidales | 5.43E-04 | Lachnospiraceae | 9.14E-05 | Bacteroides | 1.07E-04 |
| Alphaproteobacteria | 3.46E-04 | Lactobacillales | 5.31E-03 | Burkholderiaceae | 2.67E-02 | Streptococcus | 8.35E-03 |
| Deltaproteobacteria | 1.33E-06 | Propionibacteriales | 3.60E-04 | Bacteroidaceae | 1.07E-04 | Sphingomonas | 3.16E-03 |
| Gemmatimonadetes | 1.93E-06 | Sphingomonadales | 7.13E-04 | Streptococcaceae | 1.15E-02 | Bacillus | 1.09E-02 |
| Thermoleophilia | 6.27E-05 | Micrococcales | 2.81E-03 | Sphingomonadaceae | 7.13E-04 | Escherichia-Shigella | 2.63E-03 |
| Acidimicrobiia | 1.08E-05 | Rhizobiales | 1.23E-02 | Muribaculaceae | 6.04E-04 | Finegoldia | 6.35E-03 |
| Mollicutes | 2.39E-02 | Chitinophagales | 2.13E-05 | Family_XI | 3.40E-03 | Anaerococcus | 2.13E-04 |
